# Evc2 is a positive modulator of Hedgehog signalling that interacts with Evc at the cilia membrane and is also found in the nucleus

**DOI:** 10.1186/1741-7007-9-14

**Published:** 2011-02-28

**Authors:** Helen J Blair, Stuart Tompson, Yu-Ning Liu, Jennifer Campbell, Katie MacArthur, Chris P Ponting, Victor L Ruiz-Perez, Judith A Goodship

**Affiliations:** 1 Institute of Human Genetics, Newcastle University, Centre for Life, Central Parkway, Newcastle Upon Tyne, NE1 3BZ, UK; 2 Medical Genetics Institute, Cedars-Sinai Medical Center 8727 West Third Street, Suite 203, Los Angeles, CA 90048, USA; 3 MRC Functional Genomics Unit, University of Oxford, Department of Physiology, Anatomy and Genetics, South Parks Road, Oxford, OX1 3QX, UK; 4 Instituto de Investigaciones Biomédicas de Madrid (CSIC-UAM) and Ciber de enfermedades raras (Ciberer), Arturo Duperier, 4, 28029, Madrid, Spain

## Abstract

**Background:**

Evc is essential for Indian Hedgehog (Hh) signalling in the cartilage growth plate. The gene encoding Evc2 is in close proximity in divergent orientation to *Evc *and mutations in both human genes lead to the chondrodysplasia Ellis-van Creveld syndrome.

**Results:**

Bioinformatic analysis reveals that the *Evc *and *Evc2 *genes arose through a duplication event early in metazoan evolution and were subsequently lost in arthropods and nematodes. Here we demonstrate that Evc2 is essential for Hh pathway activation in response to the Smo agonist purmorphamine. A yeast two-hybrid screen using Evc as bait identified Evc2 as an Evc binding partner and we confirmed the interaction by immunoprecipitation. We developed anti-Evc2 antibodies and show that Evc2 and Evc co-localize at the basal body and also on primary cilia. In transfected cells, basal body and cilia localization is observed when Evc and Evc2 constructs are co-transfected but not when either construct is transfected individually. We show that Evc and Evc2 are cilia transmembrane proteins, the C-terminus for both being intracellular and Evc2, but not Evc, having an extracellular portion. Furthermore, Evc is absent at the basal body in Evc2 null cells. Using Western blots of cytoplasmic and nuclear protein, we also demonstrate that full length Evc2 but not Evc, is located in the nucleus.

**Conclusions:**

We demonstrate for the first time that Evc2 is a positive regulator of the Hh signalling pathway and that it is located at the basal body of primary cilia. We show that the presence of Evc and Evc2 at the basal body and cilia membrane is co-dependent. In addition, Evc2, but not Evc, is present in the cell nucleus suggesting movement of Evc2 between the cilium and nucleus.

## Background

*EVC *was first identified through a positional cloning approach as the gene defective in patients with the recessive disorder Ellis-van Creveld syndrome (EvC) [1]. The consistent features of this condition are short ribs, short limbs, postaxial polydactyly, dental abnormalities and nail dysplasia. Failure to find *EVC *mutations in a number of consanguineous EvC families mapping to the same region of chromosome 4 led to investigation of other genes in the EvC critical interval and to the identification of mutations in a second gene, *EVC2*, which is in close proximity to *EVC *[2]. The two genes are in divergent orientation with their translational start sites separated by only 2.86 kb in the human genome [3] and 1.75 kb in the mouse [4]. The phenotype associated with mutations in either of the two genes is indistinguishable indicating that *EVC *and *EVC2 *act in a common pathway [5,6].

Mice lacking Evc, like patients with Ellis-van Creveld syndrome, have short limbs, short ribs and dental abnormalities. On histological analysis of the growth plate, they have epiphyseal shortening and defective periosteal induction compatible with a defect in Indian Hedgehog (*Ihh*) signalling. Studying expression of *Ihh *and its downstream targets by *in situ *hybridization demonstrated normal *Ihh *expression but diminished mRNA levels of the Ihh downstream targets, *Patched1 *(*Ptch1)*, *Gli1 and Pthrp*. *In vitro *studies treating mouse embryonic fibroblasts (MEFs) and chondrocytes with the Hedgehog (Hh) agonist purmorphamine confirmed that Hh signal transduction is defective in cells lacking Evc [7]. Thus Evc is essential for Ihh signalling in the cartilage growth plate.

We have previously shown that Evc localises to the base of primary cilia [7]. Seminal studies demonstrated that primary cilia are required for Sonic hedgehog (Shh) signalling [8,9] and subsequent studies have confirmed that this is also the case for Ihh signalling [10,11]. Proteins are transported from the base to the tip of the cilium by anterograde intraflagellar transport (IFT) and back to the base by retrograde IFT, outward transport being mediated by a kinesin motor and retrograde transport by a dynein motor. Key components of Hh signalling such as Ptch1 and Smoothened (Smo) have been shown to enter and leave the cilium depending on the activation status of the pathway [12]. Hh signalling is ultimately meditated by the Gli transcription factor family of proteins which are observed in the cilia as well as in the nucleus [13]. In the absence of Hh signal, Gli3 is processed into a repressor form Gli3R, transcription of Gli3 targets being dependent on the balance between activator full-length Gli3 and Gli3R. Gli3 processing is reduced both when anterograde IFT is disrupted and when retrograde IFT is disrupted [8-10,14]. In *Evc*^*-/- *^mice, in contrast to IFT mutants, Gli3 processing appears normal on protein extracts from E14.5 limbs though as with the IFT mutants, the expression of gene targets of Hh signalling such as *Ptch1 *and *Gli1 *are diminished [7]. In this study we demonstrate that, in addition to Evc, Evc2 is also required for Hh signal transduction. We have shown direct interaction between Evc and Evc2, have investigated Evc and Evc2 subcellular localisation and discuss the significance of these findings.

## Results

### Evc2 is a positive regulator of Hh signal transduction

**Figure 1:**
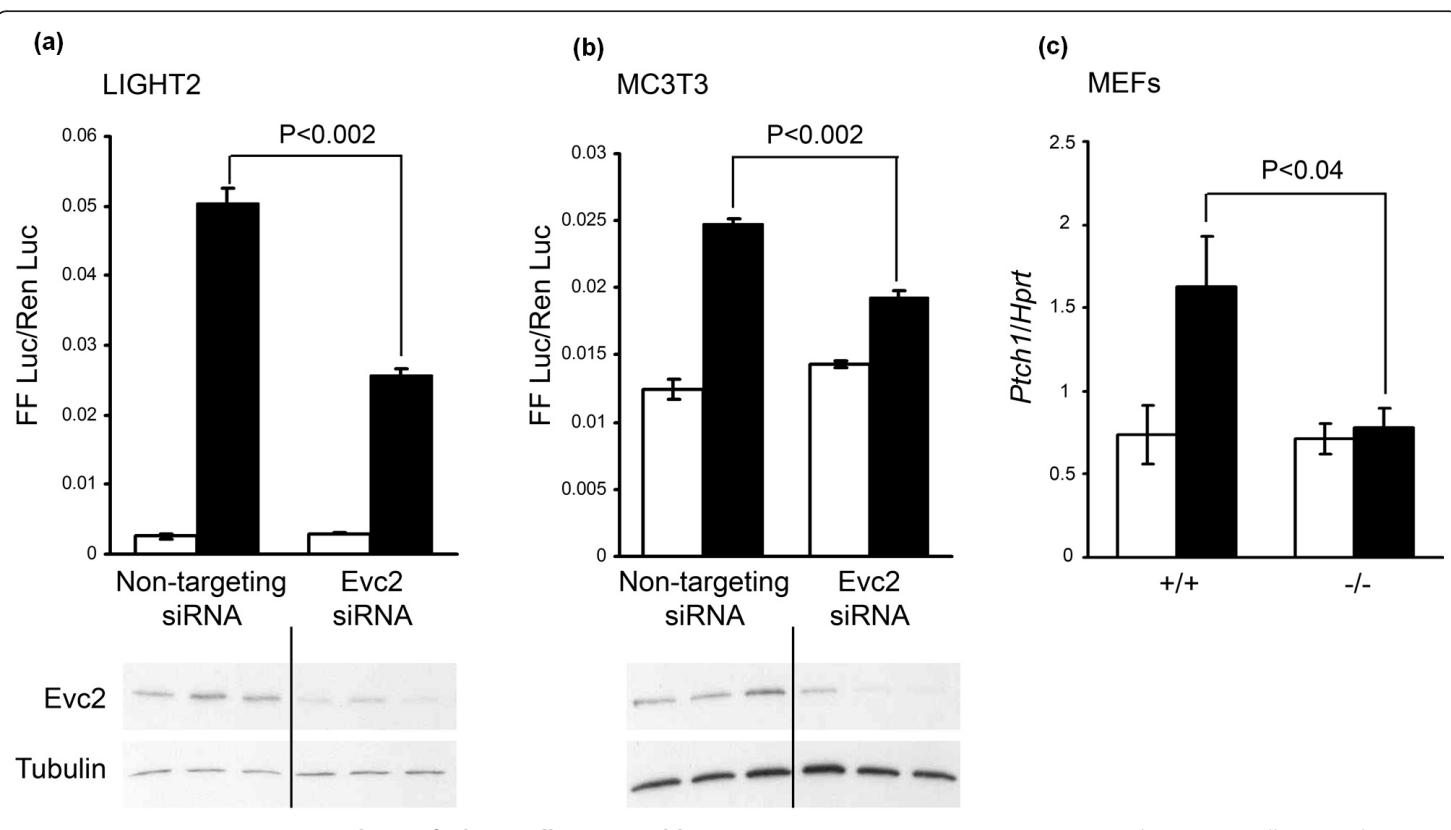
**Evc2 is a positive regulator of Hh signalling**. **(a and b)**. Representative reporter assays in LIGHT2 Gli-reporter cells (a) and MC3T3 cells (b) transfected with siRNA and treated with control DMSO (white bars) or Hh agonist purmorphamine (black bars). Purmorphamine increases expression of the Gli reporter (FF Luc) with respect to the Renilla control (Ren Luc). Evc2 knockdown was confirmed by western blotting (lower images). Evc2 (approximately 140 kDa) was detected using R1656 and α tubulin (50 kDa) was used as a loading control. Cells with reduced Evc2 protein levels (approximately 80% less than normal) have a significant reduction in Gli reporter expression in response to purmorphamine compared to those transfected with the non-targeting siRNA pool. **(c)**. The purmorphamine induced expression of the Hh responsive gene, *Ptch1*, is significantly diminished in Evc2 null MEFs (-/-) compared to wild-type controls (+/+) as assessed by RT-PCR.

Evc is known to be a positive regulator of Hh signalling both in the cartilage growth plate and in cultured chondrocytes and MEFs. The observation that *EVC *and *EVC2 *mutations are associated with the same phenotype indicates that Evc2 is likely to be a positive regulator of Hh signalling. We have tested this hypothesis using siRNA to knockdown Evc2 expression in LIGHT2, Hh reporter cells [15]. These cells are a mouse fibroblast line that stably express a Gli-dependent firefly luciferase and a TK Renilla luciferase control to allow quantitation of Hh pathway activation. LIGHT2 cells transfected with Evc2 siRNA had reduced Evc2 protein levels and had a diminished response to the Smo agonist purmorphamine compared to controls transfected with a non-targeting siRNA (Figure 1a). This result was confirmed in osteoblast-derived MC3T3 cells co-transfected with the Gli-dependent firefly luciferase and TK Renilla luciferase plasmids (Figure 1b). We also assessed expression of the Hh target gene, *Ptch1 *compared to expression of the *Hprt *housekeeping gene in Evc2 null MEFs by RT-PCR. *Ptch1 *expression in response to purmorphamine in Evc2 mutant MEFs was reduced compared to wild-type MEF controls (Figure 1c). Since purmorphamine activates Smo, these data confirm that, like Evc, Evc2 is a positive regulator of the Hh signalling pathway.

### Evc and Evc2 are homologues resulting from an ancient gene duplication

We investigated what the protein sequence of *EVC *and *EVC2 *could tell us about their relationship to each other and to their function.

Evc (a 1005 amino acid protein) has a predicted signal anchor sequence and Evc2 (a 1220 amino acid protein) has a predicted signal peptide sequence and a second predicted transmembrane region (amino acids 209 - 231), the only feature of the proteins C-terminal to these transmembrane sequences being coiled-coil regions. Sequence analysis predicts that they have resulted from an ancient duplication of a pre-existing gene whose occurrence predates the radiation of most metazoan lineages [see additional file 1]. *EVC2 *was identified as showing significant sequence similarity to *EVC *over approximately 550 amino acids when the latter was used to query the non-redundant protein sequence database using PSI-BLAST (second iteration, *E *= 6 × 10^-14^). Next, *EVC *and *EVC2 *orthologues were each found as gene predictions in the genome assemblies of amphioxus (*Branchiostoma floridae*), sea urchin (*Strongylocentrotus purpuratus*), gastropod snail (*Lottia gigantea*) and sea anemone (*Nematostella vectensis*). For example, a BLAST search of the nonredundant sequence database with sea anemone *EVC2 *as query revealed significant similarity (*E *= 6 × 10^-25^) with human *EVC2 *over 840 amino acids. Since these invertebrate species and mammals last shared a common ancestor about 670 to 820 million years ago [16], this indicates that the gene duplication giving rise to *EVC *and *EVC2 *occurred in an early metazoan species. In amphioxus, sea urchin and snail, but not sea anemone, genome assemblies these two genes, as in vertebrates, lie in close proximity in a 5' to 5' head-to-head arrangement. This arrangement, and the close proximity of their transcription start sites, suggests that *EVC *and *EVC2 *share a bidirectional promoter [17]. This, in turn, suggests that *EVC *and *EVC2 *need to be co-ordinately expressed in order to maintain an appropriate stoichiometry, or because they function in the same biological pathway. An *EVC*-like gene is also predicted in the placozoan, *Trichoplax adhaerens*, the most basal metazoan known [18] thereby confirming the presence of *EVC*-like genes in the earliest metazoans. *EVC *and *EVC2 *orthologues are absent from *Drosophila *and *Caenorhabditis elegans *genomes, indicating that they have been deleted since they last shared an ancestor with mammals.

### Direct protein interaction between Evc and Evc2

**Figure 2:**
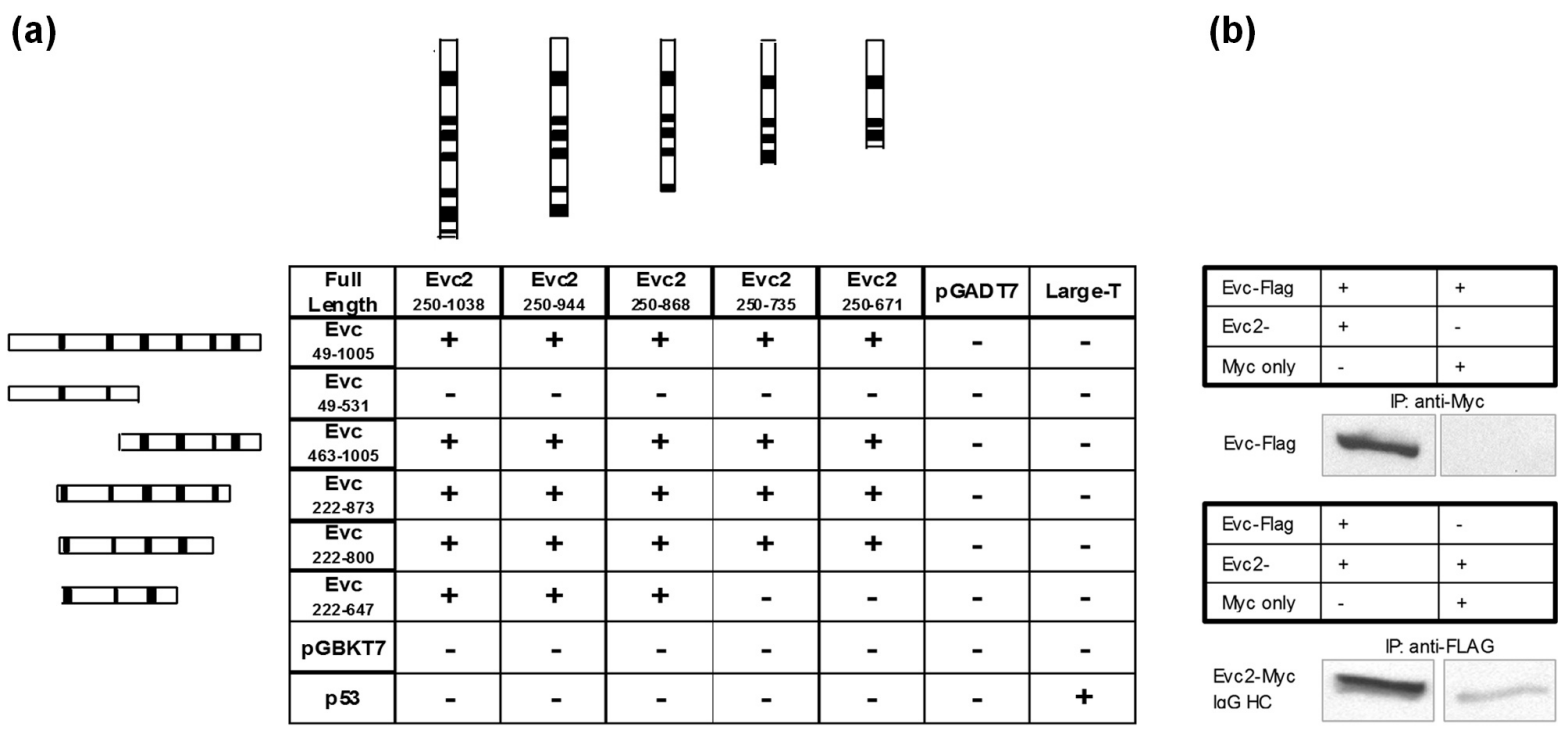
**Protein-protein interaction of Evc and Evc2**. **(a)**. Directed yeast two-hybrid assay. For each construct, the portion of Evc and Evc2 coding sequence expressed is depicted. Predicted coiled-coil regions are indicated by black boxes. Negative controls (empty vectors pGBKT7 and pGADT7) and positive controls (p53 and Large-T antigen) were included. + indicates colony growth/interaction, - indicates no colony growth/lack of interaction. **(b)**. FLAG-tagged Evc is immunoprecipitated by Myc-tagged Evc2 but not by the Myc epitope alone. Myc-tagged Evc2 is immunoprecipitated by FLAG-tagged Evc but not by the Flag epitope alone. A non-specific band corresponding to the IgG heavy chain (HC) is indicated.

To search for Evc-interacting proteins we screened a cDNA library derived from E11 mouse embryos by the yeast two-hybrid assay using *Evc *sequence encoding amino acids 49 - 1005 as bait. This screen did not identify any known Hh pathway components but did identify Evc2 as an Evc-interacting partner. In order to map this interaction, deletion constructs for both Evc and Evc2 were generated and used in a directed yeast-two-hybrid assay (Figure 2a). Significant binding was observed with the following *Evc *constructs: expressing amino acid 49 - 1005, 463 - 1005, 222 - 873 and 222 - 800 and Evc2. No growth was observed with an *Evc *construct expressing amino acids 49 - 531 and restricted growth with the construct coding for amino acids 222 - 647. Thus interaction was observed for the four constructs containing the third and fourth coiled-coil regions but restricted interaction with the construct containing only the first three coiled-coil regions and no interaction detected with the constructs containing only the first two coiled-coil regions. Whilst the fifth and sixth coiled-coil regions were contained within constructs for which interaction was observed they were not tested independently of the fourth coiled-coil region. Significant binding was observed with the *Evc2 *construct expressing amino acids 250 - 671 and Evc (Figure 2a). This portion of the Evc2 protein contains the first three predicted coiled-coil regions; interaction was not assessed for these three coiled-coil regions independently. We attempted to express full-length Evc and Evc2 proteins in mammalian cells to test for their interaction by co-immunoprecipitation (Co-IP). Full-length proteins could not be expressed at high levels in mammalian cells and were insoluble in standard Co-IP buffers. Therefore we co-expressed the shortest constructs for which strong interaction was observed (Evc amino acids 463 - 1005 and Evc2 amino acids 250 - 671) in HEK 293 cells with Flag and Myc-tags, respectively, to test whether Evc and Evc2 interact in a mammalian expression system. HEK 293 cells are ciliated renal cells [19]. Using antibodies against the tags, each protein was bound independently to protein G Sepharose beads. The beads were then extensively washed prior to elution of the interacting proteins and we detected co-immunoprecipitaton of the corresponding interacting partner by Western blot. Evc and Evc2 co-immunoprecipated with each other but not with vector or antibody controls confirming their interaction (Figure 2b).

### Evc and Evc2 colocalize at the basal body and cilia

**Figure 3:**
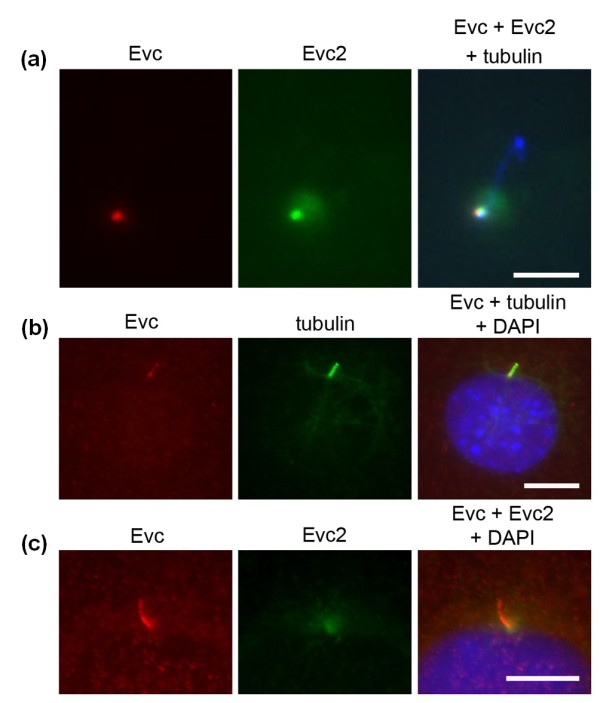
**Subcellular localization of native Evc and Evc2**. **Each row consists of images from one representative cell. (a)**. Evc (S43B; red) and Evc2 (R1656; green) colocalize at the base of the primary cilium in an IMCD3 cell. γ- and acetylated tubulin antibodies (both blue) identify the centrioles and the primary cilium respectively. Evc2 has an additional pericentriolar distribution. Scale bar 5 μm. **(b)**. Evc is located along the length of the ciliary axoneme in MC3T3 cells. Evc (S43B; red) colocalizes with the primary cilia marker acetylated tubulin (green). Scale bar 10 μm. **(c)**. Evc2 is concentrated at the base of the primary cilium and only partially colocalizes with Evc in MC3T3 cells. Evc (S43B; red) and Evc2 (R1656; green). Scale bar 10 μm.

**Figure 4:**
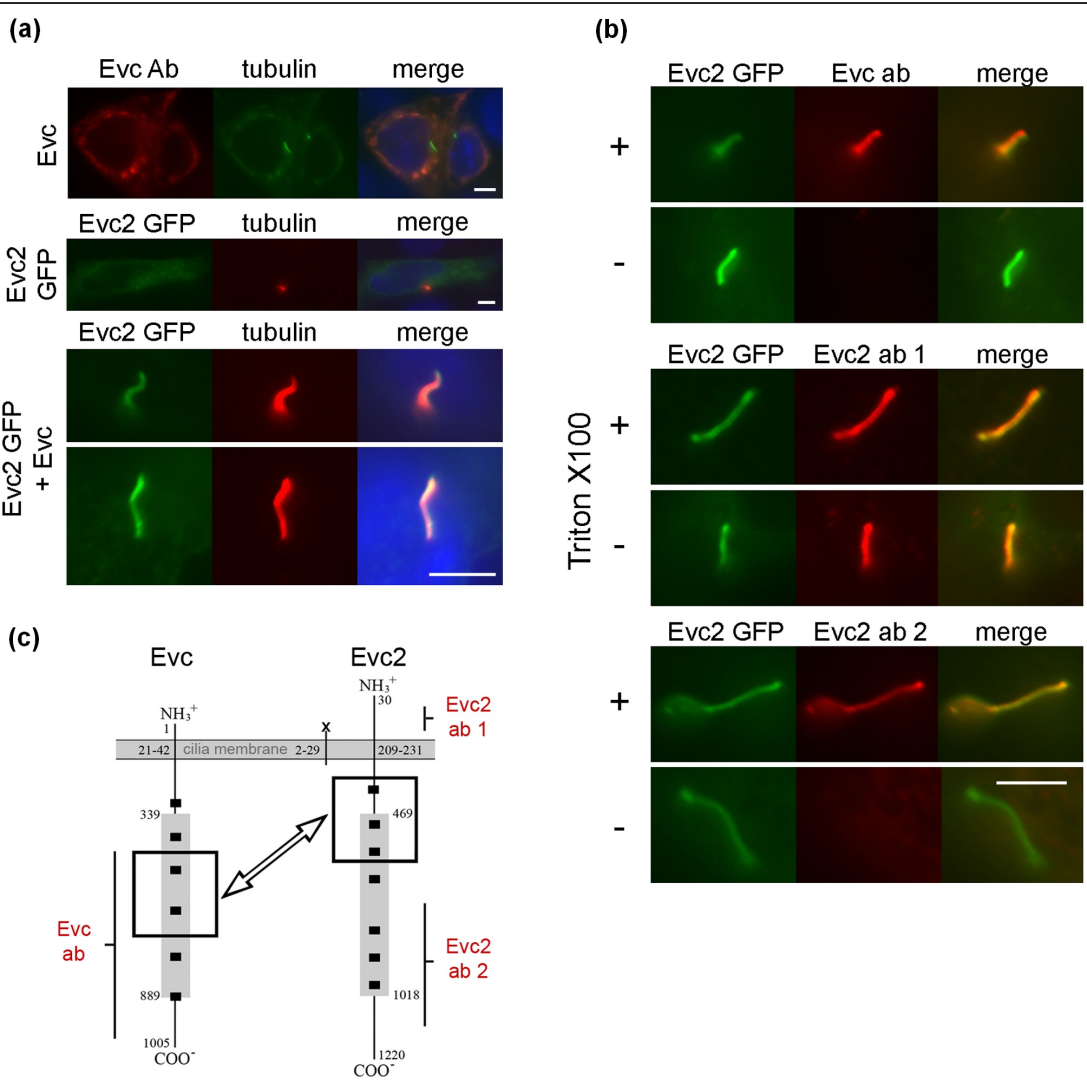
**Evc and Evc2 localize to the ciliary membrane in co-transfected IMCD3 cells**. **Each row consists of images from one representative cell. (a)**. Single transfection and co-transfection of Evc and Evc2-GFP constructs (the protein expressed is given on the left of each image). Expression of either protein alone does not result in localization on cilium. Evc is identified by S43B immunostaining (Evc ab, red), Evc2 by the GFP tag and cilia by acetylated tubulin staining (tubulin; green or red). Co-expression of both proteins results in their expression on the cilium. (cilia from two independent cells are shown). Merged images also show DAPI staining (blue). **(b)**. Immunodetection of Evc and Evc2-GFP co-expressed in cells treated with (+) or without (-) Triton X100 to permeabilize the membranes and allow access to the antibodies. Evc ab (S43B), raised to Evc aa 459 - 999, does not detect Evc in non-permeabilized cells; Evc2 ab 1 (Y20), raised to a Evc2 peptide between aa 50 - 100, detects Evc2 GFP in non-permeabilized cells and Evc2 ab 2 (R1656), raised to Evc2 aa 780 - 1124, does not detect Evc2 in non-permeabilized cells. **(c)**. Schematic representation of Evc and Evc2 on the ciliary membrane as determined from B, the C-termini of both proteins are intracellular. The regions for which we confirmed interaction are indicated by boxes and the predicted coiled-coil regions are shown as small black squares. Regions that are homologous (EVC: 339 - 889, and EVC2: 469 - 1018) are indicated by shaded rectangles. The epitope regions detected by Evc and Evc2 antibodies are indicated. Scale bar throughout 5 μm.

We have previously observed Evc at the distal end of the basal body *in vivo *and *in vitro *[7]. To study Evc2 localization we raised a new polyclonal anti-Evc2 antibody, R1656, and we observed Evc2 co-localization with Evc at the basal body in fibroblast, osteoblast and chondrocyte cultures and in renal derived IMCD3 cells (Figure 3a and not shown). In addition, we observed diffuse pericentriolar staining for Evc2 (Figure 3a). Specificity of the Evc2 immunostaining was demonstrated by antigen blocking and immunostaining in null Evc2 MEFs (data not shown). On testing additional cell lines to ascertain Evc/Evc2 localization we observed Evc along the length of the ciliary axoneme in all cilia of osteoblast-derived MC3T3 cells (Figure 3b). In these cells co-staining detected Evc2 mainly at the base of cilia (Figure 3c). Co-transfection of *Evc *and *Evc2-GFP *constructs into IMCD3 cells detected both proteins along the length of the cilium (Figure 4a-b), supporting the observations in MC3T3 cells. Co-transfection into MC3T3 and NIH3T3 cells also resulted in both proteins localizing to the cilia (results not shown). However, we did not observe basal body or cilia localization when transfecting constructs expressing either tagged Evc or Evc2 individually (Figure 4a).

### Evc and Evc2 topography in the cilia membrane

Evc2 has a predicted signal peptide sequence and a second predicted transmembrane region (amino acids 209 - 231). To address whether Evc2 spans the cilia membrane and demonstrate its orientation in the membrane we immunostained co-transfected, non-permeabilized IMCD3 cells with antibodies generated against peptides N-terminal and C-terminal of the second transmembrane domain (Figure 4b). Y-20 antibody was raised against a peptide near the N-terminus of Evc2 between the two predicted transmembrane domains and R1656 against a C-terminal region of Evc2 (amino acids 780 - 1124) (Figure 4c, Evc2 ab 1 and 2, respectively). Y-20 detected Evc2-GFP along the length of the cilium in both permeabilized and non-permeabilized cells demonstrating that this portion of Evc2 is extracellular (Figure 4b, Evc2 ab1). However, R1656 only detects cilial Evc2-GFP in permeabilized cells, indicating that the region that is C-terminal of the transmembrane domain and detected by this antibody is intracellular (Figure 4bc Evc2 ab 2). S43B antibody (raised against Evc amino acids 459 - 999) detects native Evc in cilia in permeabilized MC3T3 cells, but does not detect Evc in non-permeabilized MC3T3 cells (result not shown) or in non-permeabilzed co-transfected IMCD3 cells (Figure 4bc, Evc ab). This indicates that the region of Evc C-terminal of the transmembrane domain is also intracellular. Thus, the regions of Evc and Evc2 that we have shown by Co-IP to interact are intracellular whilst the N-terminus of Evc2 is extracellular (Figure 4c).

### Evc2 is required for localization of Evc at the base of primary cilia

**Figure 5:**
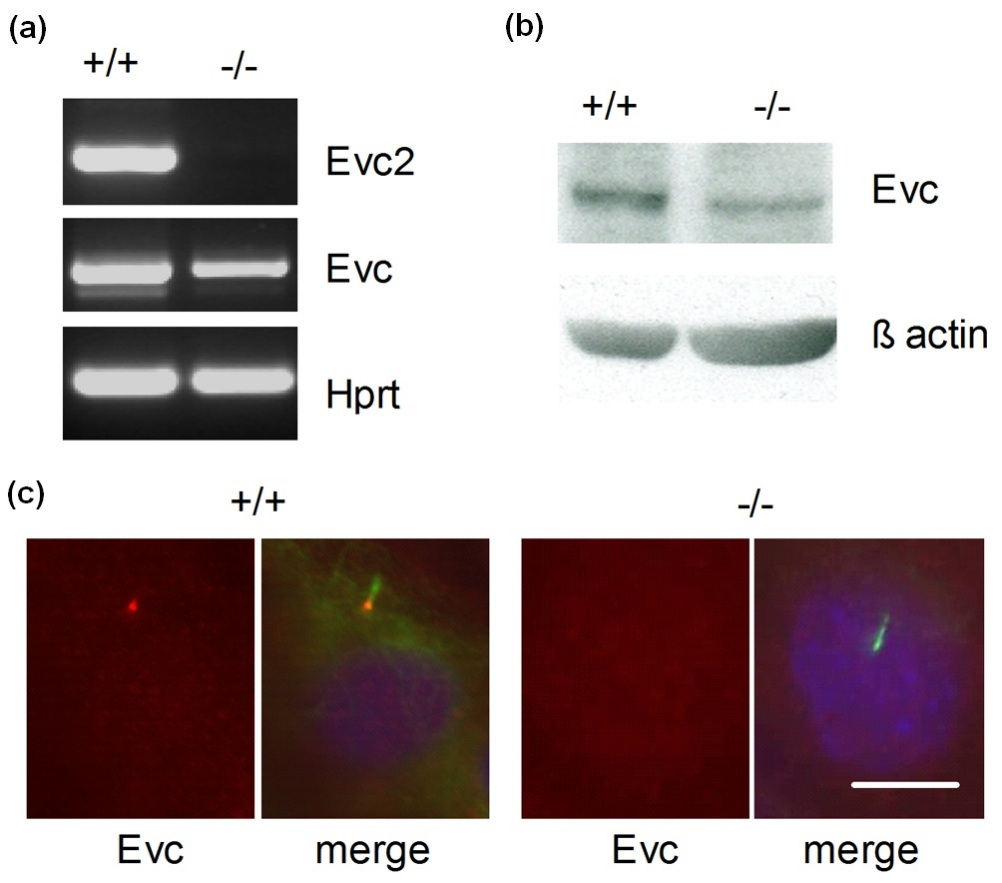
**Evc2 is required for the localization of Evc at the base of primary cilia**. **(a)**. RT-PCR amplification products from Evc2 null (-/-) and wild-type (+/+) MEFs. As expected, no Evc2 transcript was detected in the Evc2 null MEFs. A significant amount of Evc transcript was amplified in the Evc2 null MEF sample. Hprt transcript was amplified as a control. **(b)**. Western blot analysis of Evc protein in Evc2 null MEFs. The amount of β-actin detected on the same blot was used as a loading control. Evc (approximately 130 kDa) is present in Evc2 null (-/-) MEFs despite having reduced levels (approximately 50%) **(c)**. Representative immunofluorescent staining of Evc in MEF cells. Despite the presence of protein, Evc (red) was not detected at the base of primary cilia in Evc2 null MEFs (-/-). Primary cilia were identified by the presence of acetylated tubulin (green) and nuclei stained with DAPI (blue). Scale bar 10 μm.

**Figure 6:**
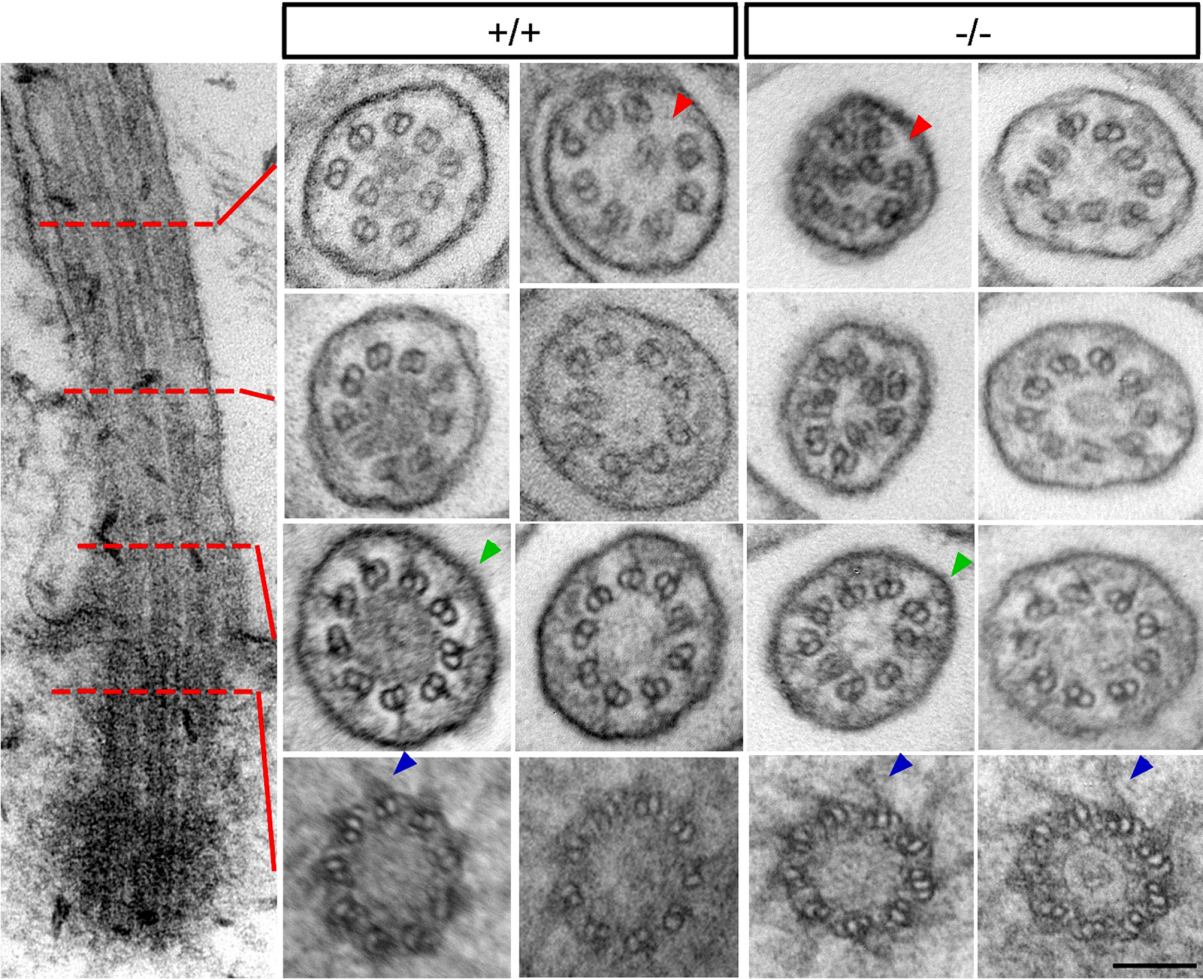
**Longitudinal and transverse sections of chondrocyte cilia obtained by transmission electron microscopy**. The longitudinal section and red lines (left) indicate the approximate position of transverse sections. Transverse sections of cilia are from wild-type (+/+) and Evc mutant (-/-) chondrocytes cells. In both genotypes, disorganized doublets were found in the distal region (red arrow heads) and the champagne glass structures were found in the proximal region (green arrow heads). Nine triplet microtubules and spike structures (blue arrow heads) could be identified in transverse sections of basal body. Scale bar 100 nm.

Since both Evc and Evc2 are co-dependent for cilia localization of expressed proteins, we tested if Evc2 is required for cilia localization of Evc in Evc2 null cells. MEFs derived from Evc2 mutant mice do not produce any Evc2 transcript or protein (Figure 5a and 6a). Evc and Evc2 are both detected at the base of primary cilia in wild-type MEFs (Figure 5c and not shown, respectively). Despite the presence of Evc transcript and protein (Figure 5a-b) Evc was not detected at the base of cilia in Evc2 null MEFs. This confirms that Evc2 is essential for Evc cilia localization.

### Primary cilia of cells lacking Evc appear structurally normal

Although cilia in mice lacking Evc look normal on standard microscopy [7] the possibility remained that the Hh signalling defect could be secondary to a structural abnormality of cilia. We therefore examined cilia structure in chondrocytes using transmission electron microscopy (TEM). In the basal body region, cilia from mutant chondrocytes showed the normal configuration of 9 triplet microtubules with transition fibers radiating from the triplets at the distal end of the basal body (Figure 6, blue arrowheads). The champagne glass structures that connect the microtubule doublets to the ciliary membrane seen in the transition zone [20] are present in the mutant chondrocytes (Figure 6, green arrowheads). Sections through the mid-portion of the cilia show 9 microtubule doublets with normal orientation and structure. In the distal region of the cilia there is some collapse of the microtubule ring in chondrocyte cilia from both wild-type and *Evc*^*-/- *^mouse (Figure 6, red arrowheads), a feature that has been reported before in normal cilia [21,22]. All observed cilia showed complete triplet microtubule structure in the basal body and doublet microtubules in the ciliary region. No structural differences were observed between the cilia from wild-type and *Evc*^*-/- *^chondrocytes.

### Evc2, but not Evc, is found in the nucleus

**Figure 7:**
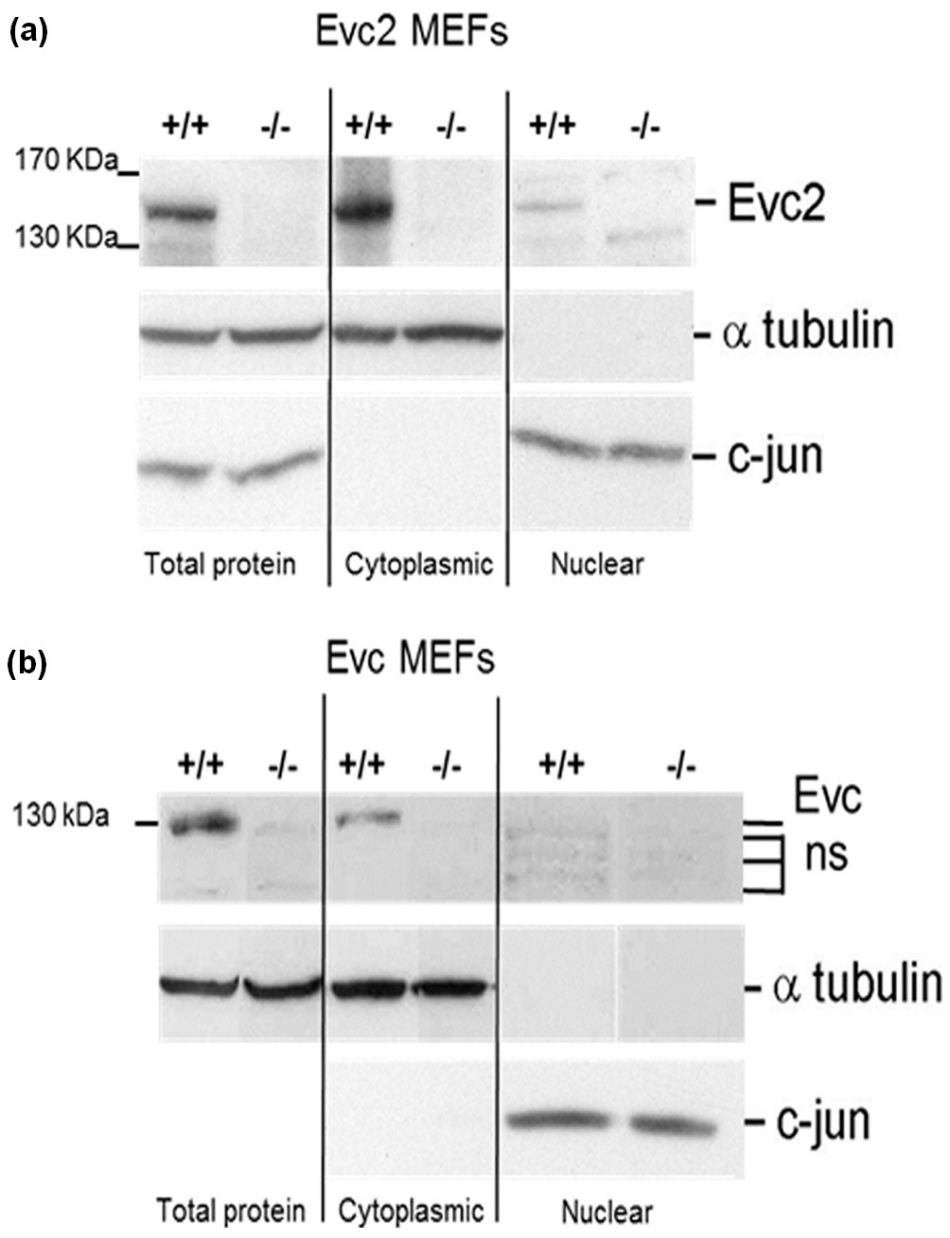
**Evc2 is present in both the cytoplasm and the nucleus of MEF cells**. Western blot analysis of total, cytoplasmic and nuclear proteins from wild-type (+/+) and Evc2 (-/-) (**a**) and Evc (-/-) (**b**) MEFs. The purity of the cytoplasmic and nuclear protein samples was confirmed using antibodies to α-tubulin (approximately 50 kDa) exclusively cytoplasmic and c-Jun (approximately 43 kDa), exclusively nuclear. Full-length Evc2 (140 kDa) was detected in both the cytoplasmic and nuclear samples. Full-length Evc protein (130 kDa) was only detected in the cytoplasm. Antibodies to both proteins detected several additional bands that are assumed to be non-specific as they are present in the null MEFs.

Evc and Evc2 are detected on cilia in several different cell types by immunostaining. To determine if Evc and Evc2 are located elsewhere in the cell we performed subcellular fractionation on MEFs derived from wild-type and null mice. The purity of cytoplasmic and nuclear protein samples was confirmed using antibodies to alpha tubulin and c-jun, respectively (Figure 7). Specificity of the antibodies to Evc and Evc2 was confirmed by the absence of bands on Western blots of MEFs derived from null mice. We detected full-length Evc2 (approximately 140 kDa protein) both in the cytoplasmic and in the nuclear fractions but only detected full-length Evc (approximately 130 kDa protein) in cytoplasmic fractions.

## Discussion

The fact that mutations in *EVC *and *EVC2 *cause the same human phenotype, the chondrodysplasia Ellis-van Creveld syndrome, suggested that these two proteins act in the same pathway. We have shown that Evc and Evc2 are both positive regulators of the Hh pathway and that they interact directly with each other. The Hh pathway defect described in Evc and Evc2 mutants is downstream of the action of purmorphamine. It is known that purmorphamine directly activates Smo and promotes its entry onto the cilium [23,24]. Thus, both Evc and Evc2 are positive regulators that act on, or downstream of, Smo in Hh signal transduction.

Analysis of *EVC *and *EVC2 *gene sequences predicts that they have arisen from an ancient duplication. Conservation of their 5' to 5' head to head arrangement and proximity of transcriptional start sites suggests that *EVC *and *EVC2 *need to be co-ordinately expressed in order to maintain an appropriate stoichiometry, or because they function in the same biological pathway. It is interesting to note that *Evc*-like genes are absent from organisms that do not use cilia for Hh signalling (flies) or that do not have Hh signalling (worms).

We studied Evc and Evc2 subcellular localization and observed that Evc2 co-localizes with Evc at the base of primary cilia in fibroblast and chondrocyte cultures and in renal-derived cell lines. On testing additional cell lines we observed additional Evc along the length of the ciliary axoneme in the osteoblast-derived MC3T3 cell line. One interpretation of the difference between the immunofluorescence analysis in this cell line and other cell lines, primary cell cultures and on tissue cryosections is that the amount of native protein normally present in cilia is below the sensitivity of immunofluorescence in most cells. Indeed, MC3T3 cells do express higher levels of Evc than other cells studied (Western blot analysis not shown). Cilia localization of co-expressed protein from constructs is in keeping with this interpretation but another possibility is that localization of Evc and Evc2 to the cilia is dependent on additional factors, analogous to the way in which localization of Ptch1, the Hh receptor, and Smo localization changes on addition of ligand. Ptch1 localizes to the cilium in the absence of ligand, but on ligand binding leaves the cilium upon which Smo enters the cilium and in turn regulates processing and activation of the three Gli transcription factors. We did not observe a change in Evc or Evc2 localization in MC3T3 and IMCD3 cells after treatment with purmorphamine (results not shown) which suggests that cilia localization of Evc and Evc2 is not dependent on pathway activation status.

The observation that overexpressed Evc and Evc2 proteins localize to the cilium after co-transfection, but not when expressed individually, suggests that basal body and cilia localization is dependent on the interaction between Evc and Evc2. This was confirmed using MEFs from Evc2 null mice where Evc is present but does not localize to the base of primary cilia. Co-dependent localization of Evc and Evc2 could explain why mutations in either gene result in an indistinguishable patient phenotype. Given that both Evc and Evc2 possess transmembrane domains, observing them on the cilium led us to question whether they span the cilia membrane and, if so, their orientation in the membrane. We addressed this by comparison of immunofluorescence on permeabilized and non-permeabilized cells with antibodies generated against peptides N-terminal and C-terminal of the transmembrane domain of Evc2. These confirmed that Evc2 spans the cilia membrane and that the N-terminal portion is extracellular. Evc has a predicted signal anchor sequence and comparison of immunofluorescence signal on permeabilized and non-permeabilized cells demonstrated that Evc is intracellular. Furthermore, the regions of Evc and Evc2 that we have demonstrated to interact are both intracellular (Figure 4c).

A question that arises when a Hh signalling defect results from loss of a cilia protein is whether this defect is due to a cilia abnormality or due to specific modulation of Hh signal transduction. Ultrastructural analysis of cilia of Evc mutant cells does not revealed any abnormalities suggesting that the mutant phenotype does not result from defective cilia but rather that Evc is a specific modifier of Hh signal transduction.

In addition to the cilia localization, we detected Evc2 in MEF nuclear extracts by Western blotting. We detected Evc in cytoplasmic but not in nuclear extracts suggesting that Evc does not enter the nucleus, although we cannot exclude the possibility that Evc is present at undetectable levels in the nucleus. Evc and Evc2 were not observed in the nucleus by immunostaining, but a diffuse nuclear distribution may be difficult to distinguish from background staining. Also, overexpressed proteins were not observed in the nucleus when expressed alone or when co-expressed leading us to conclude that additional factors are required to transport Evc2 into the nucleus, a process that may depend on activation of the Hh pathway.

The presence of Evc2 both in the ciliary membrane and in the nucleus is intriguing. There is a precedent for proteolytic processing and translocation to the nucleus of the C-terminal region of a cilia membrane protein in polycystin 1 (PC1) [25]. After cleavage, the C-terminal peptide of PC1 moves to the nucleus where it associates with Stat6 and p100 to activate gene transcription. The nuclear localization and the N-terminal extracellular portion of Evc2 may indicate an analogous role for Evc2 in the regulation of Hh target gene transcription in response to extracellular signals.

One of the many recent surprises regarding Hh signalling was the finding that the Gli transcription factors localize to cilia as well as the nucleus. Further experiments are required to determine whether Ellis-van Creveld proteins move in conjunction with Gli proteins or via an independent pathway.

## Conclusions

We have previously shown that Evc is a positive modulator of Ihh signalling at the growth plate acting at or downstream of Smo. Here we have shown that Evc2 is also required for Hh signalling and that there is a direct physical interaction between the two proteins.

We have shown that they localize to the membrane of primary cilia in a co-dependent manner and have deduced their orientation within the cilia membrane, the C-terminus of each being intracellular and only Evc2 having an extracellular portion. We have found that Evc2 is present in the nucleus but cannot detect Evc in nuclear extracts. We conclude that Evc and Evc2 are interacting proteins that together modulate Hh signal transduction.

## Methods

### Cell culture

All cells were cultured in medium containing 10% FBS (Invitrogen Ltd; Carlsbad, CA, USA) at 37°C and 5% CO_2. _Medium was obtained from Invitrogen Ltd. LIGHT2 cells were cultured in DMEM with 4.5 g/l glucose; MC3T3 cells in alpha MEM and IMCD3 and HEK 293 cells in DMEM/F12 (1:1) and DMEM respectively with non-essential amino acids. Mouse embryonic fibroblasts (MEFs) were established from E14.5 mutant and littermate control embryos [26] and cultured in DMEM without Na pyruvate, with non-essential amino acids and penicillin/streptomycin. Evc mice and the generation of *Evc*^-/- ^MEFs was described previously [7]. *Evc2*^-/- ^MEFs were derived from Evc2 null mice that were generated by replacing exon1 of *Evc2 *with a reporter gene encoding the green fluorescence protein fused in frame to the first ATG of *Evc2 *(unpublished data). Experiments were performed using MEFs cultured for less than eight passages.

### SiRNA knock-down and Hh assays

siRNAs were ON-TARGETplus SMARTpool for Mouse *Evc2 *and ON-TARGETplus non-targeting siRNA pool 1 as a control (Dharmacon). Cells were transfected in triplicate on 12 well plates when 50% confluent. LIGHT2 cells were transfected with 100 pmoles siRNA/well using Dharmafect1 reagent (Dharmacon). MC3T3 cells were transfected with 80 pmoles siRNA/well using X-tremeGENE siRNA reagent (Roche Applied Science, Penzberg, Germany). One day later MC3T3 cells were co-transfected with the 8xGli-BS-Luc [27] and TK-Renilla plasmid (Promega WI, USA) at a ratio of 4:1 using Fugene HD reagent (Roche Applied Science, Penzberg, Germany). Cells were treated for 48 hours with purmorphamine (2 μM, Calbiochem, San Diego, CA, USA) or an equivalent amount of DMSO carrier as a negative control. LIGHT2 and MC3T3 cells were harvested 72 and 48 hours after transfection, respectively, and assayed for luciferase reporter expression using the Dual Luciferase Reporter assay system (Promega WI, USA) and a Luminoskan Ascent luminometer (Thermo Scientific, Walthem, MA, USA). The data was normalized by calculating the ratio of Firefly to Renilla luciferase readings (FF Luc/Ren Luc). Each experiment was repeated at least twice in triplicate. *P *values were calculated by *t*-test (Two Sample Assuming Unequal Variances). Evc2 protein knockdown in cell lysates was assessed by Western blotting and densitometry.

For *Ptch1 *RT-PCR in MEFs, assays were carried out in triplicate on 2 *Evc2 *^*-/- *^and 2 *Evc2 *^*+/+ *^wild-type MEF cultures. Cells were treated with purmorphamine or DMSO as above for 48 hours. RNA was prepared using Trizol reagent (Invitrogen Ltd; Carlsbad, CA, USA) and first strand cDNA was synthesized using Superscript III (Invitrogen Ltd; Carlsbad, CA, USA). Simultaneous PCR amplification of *Ptch1 *(nt 1944 - 2303 [GenBank: NM_008957]) and *Hprt *(nt 108 - 294 [GenBank: NM_013556) was performed for 22 cycles in standard PCR conditions. Ratios of *Ptch1 *to *Hprt *band intensity were determined for each culture before and after treatment.

### Sequence analysis

PSI-BLAST [28] searches employed default parameters, and mouse sequences as queries (unless otherwise stated) against the non-redundant protein sequence database held at the National Center for Biotechnology Information (Bethesda, MD). BLASTp and TBLASTn searches of *Lottia gigantea *and *Branchiostoma floridae *gene models and genome assemblies used web-based searches at the Joint Genome Institute http://www.jgi.doe.gov/. Signal peptides and anchors were predicted using SignalP-HMM [29]. Coiled coil sequences were predicted using Coils [30] and a threshold of *p *> 0.5. The phylogenetic tree of *EVC *and *EVC2 *sequences was constructed with the Fitch-Margoliash algorithm using a Poisson genetic distance and global optimization with bootstrapping (PMID: 5334057).

### Yeast Two-Hybrid analysis

Mouse *Evc *in pAS2-1 vector was transformed into yeast strain AH109 and used as a bait to screen approximately 1.25 × 10^6 ^clones from a mouse 11-day embryo cDNA library constructed in the pACT2 vector and pre-transformed into yeast strain Y187 (Clontech, Mountain View, CA, USA). Positive interactions were identified by growth of mated bait and library cells on media lacking leucine, tryptophan, histidine and adenine at 30°C for 4 - 8 days. Positive colonies were confirmed by X-alpha-galactosidase activity assay. For the directed yeast two-hybrid studies, AH109 and Y187 yeast strains were transformed with *Evc *and *Evc2 *constructs and mated. Matings between yeast containing the pGBKT7-p53 and pGADT7-T-antigen vectors were used as a control for positive interaction.

### Constructs

For yeast two-hybrid library screening, mouse *Evc *sequence encoding amino acids 49 - 1005 which does not include the transmembrane domain was cloned into pAS2-1 vector (Clontech, Mountain View, CA, USA). For directed yeast two-hybrid analysis, mouse *Evc *fragments were cloned into pGBKT7 vector (Clontech, Mountain View, CA, USA) and mouse *Evc2 *fragments were cloned into pGADT7 vector (Clontech, Mountain View, CA, USA). For co-immunoprecipitation studies, the *Evc *fragment (amino acids 463 - 991) was cloned into pCMV-3xFLAG-10 vector (3 × Flag fusion; Sigma-Aldrich, St. Louis, MO, USA) and the *Evc2 *fragment (amino acids 250 - 671) into pCMV-3 vector (Myc fusion; Stratagene Corp; La Jolla, CA. USA). For subcellular localization studies, the complete mouse Evc coding region was cloned into pcDNA3.1(-) (Invitrogen Ltd; Carlsbad, CA, USA). The complete mouse Evc2 coding region was cloned into pEGFP-N1 (Clontech, Mountain View, CA, USA). The stop codon was mutated to allow translational read-through into the EGFP gene. All constructs were sequenced to confirm correct gene sequence and reading frame.

### Co-immunoprecipitation (Co-IP)

HEK 293 (Human Embryonic Kidney) were transiently transfected with the Myc- and 3 × FLAG-tagged constructs using GeneJammer reagent (Stratagene Corp; La Jolla, CA. USA), following the manufacturer's instructions. Transfections were performed in T75 flasks at 80% confluence, using 60 μl GeneJammer and 10 μg each plasmid, and were allowed to grow for 48 hours. Cells were resuspended in lysis buffer (50 mM HEPES pH7.4, 100 mM NaCl, 100 mM EDTA, 20 mM beta-glycerophosphate, 0.5% NP-40, 1 mM PMSF, Complete Protease Inhibitor Cocktail (Roche Applied Science, Penzberg, Germany) for 30 min, and spun in a microcentrifuge for 15 min at 4°C. Lysates were incubated for 1 hour at 4°C with Protein G Sepharose 4 Fast Flow beads (GE Healthcare, Uppsala, Sweden) to pre-clear, and spun in a microcentrifuge for 10 min at 4°C. Lysates were incubated 24 hours at 4°C with Protein G Sepharose 4 Fast Flow beads (GE Healthcare, Uppsala, Sweden) and 1 μg anti-Myc (9E10; Santa Cruz Biotech Inc; CA, USA) or anti-FLAG antibody (M2; Sigma-Aldrich, St. Louis, MO, USA). The beads were then washed extensively with lysis buffer. The co-immunopreciptates were analyzed by SDS-PAGE and Western blotting with anti-Myc (Santa Cruz Biotech Inc; CA, USA) or anti-FLAG (Sigma-Aldrich, St. Louis, MO, USA).

### Evc2 antibody production

Amino acids 780 - 1124 of the mouse Evc2 protein (GenBank BAC06589) were expressed with a 6 × His tag in *E. coli*, purified by Ni^2+ ^chelation chromatography (Novagen) and used to immunize rabbit. Total IgGs were prepared from final serum (Protein G HiTrap, GE Healthcare, Uppsala, Sweden). Total rabbit IgGs often bind non-specifically to centrosomes therefore it was important that mouse Evc2 specific IgGs were isolated. For this, the antigen region was expressed in *Escherichia.coli *with a GST tag and purified on Glutathione sepharose 4B (GE Healthcare, Uppsala, Sweden). Specific anti-Evc2 IgGs, henceforth referred to as R1656, were purified by affinity to the GST-tagged Evc2 protein.

### Immunofluorescent staining

Cells were fixed in 4% (w/v) paraformaldehyde (PFA) in PBS at 4°C for 10 minutes and permeabilized in 0.1% Triton X100 in PBS for 10 minutes (S43B and permeabilization experiments) or in PBS for 10 minutes (non-permeablization experiments); ice-cold MeOH/Acetone (1:1) for 6 minutes (R1656) or ice-cold methanol for 3 minutes (Y20). Primary antibodies were: sheep polyclonal anti-Evc (S43B [7]); rabbit polyclonal anti-Evc2 (R1656); goat polyclonal anti-Evc2 (Y-20, Santa Cruz Biotech Inc; CA, USA); anti-acetylated tubulin (Sigma-Aldrich, St. Louis, MO, USA) and mouse monoclonal anti-gamma tubulin (Sigma-Aldrich, St. Louis, MO, USA). Secondary antibodies were: donkey anti-sheep AlexaFluor 594 (Molecular Probes, Invitrogen Ltd; Carlsbad, CA, USA); goat anti-rabbit FITC (Jackson ImmunoResearch Labs Inc; PA, USA); goat anti-rabbit Cy3 (Sigma-Aldrich, St. Louis, MO, USA); donkey anti-goat FITC (Jackson Immuno Research Labs Inc; PA, USA); rabbit anti-goat Cy3 (Sigma-Aldrich, St. Louis, MO, USA); donkey anti-mouse AMCA (Jackson ImmunoResearch Labs Inc; PA, USA); horse anti-mouse TexasRed (Vector Labs, UK) and goat anti-mouse FITC (Sigma-Aldrich, St. Louis, MO, USA). Samples were mounted in Vectashield with or without DAPI (Vector Labs, UK) and images captured on an Axioplan 2 fluorescence microscope (Zeiss). Antibody blocking experiments were performed by preincubating primary antibody with approximately 2 μg of GST-Evc2 on beads and GST control (for R1656). At least ten cilia were visualized in each experiment. MC3T3 cells and MEFs were serum starved overnight prior to immunofluorescent staining to induce approximately 60% ciliation of cells. Ciliation of IMCD3 cells approached 100% without serum starvation.

### Transmission electron microscopy (TEM)

Chondrocytes were isolated from the proximal tibial epiphyses of E18.5 wild-type and *Evc*^*-/- *^littermates. First tissue was dissected and washed in PBS. Cells were released from the extracellular matrix by sequential digestion with hyaluronidase (5 minutes, 1 mg/ml PBS), trypsin (10 minutes, 2.5 mg/ml PBS) and collagenase (5 hour, 3 mg/ml DMEM containing 10% FBS) at 37°C with constant rotation. The chondrocytes were incubated in DMEM for a maximum of seven days. We confirmed by real-time PCR that these cells retained chondrocyte expression profiles during this time period. Cells were grown on culture inserts (Nunc, Thermo Scientific, Walthem, MA, USA) and serum starved overnight prior to fixation to induce cilia production. Cells were fixed in 2% PFA/PBS at 4°C for 1 hour, dehydrated and embedded in LR White resin (EMS). Ultra thin sections (approximately 80 nm) were prepared on a RMC MT-XL ultramicrotome and stained on Pioloform filmed copper grids with 2% aqueous Uranyl Acetate and Lead Citrate (Leica UK Ltd). The ultra structure of 2 Evc^-/- ^and 3 wild type cilia was observed with a Philips CM 100 Compustage (FEI) Transmission Electron Microscope and digital images collected using an AMT CCD camera (Deben).

### Cytoplasmic/nuclear fractionation and Western blotting

*Evc2*^*-/- *^MEFs were characterized by RT-PCR amplification of Evc2 (nt 533 - 830 [GenBank: AB083066]); Evc (nt 1445 - 3060 [GenBank: AJ250841]) and Hprt (see above), and by western blotting. The cellular fractionation protocol was adapted from published methods [31]. Briefly null and control MEFs from T75 flasks were suspended in ice-cold cell swelling buffer containing 10 mM HEPES pH7.9; 10 mM KCl; 0.1 mM EDTA; 0.1 mM EGTA; 1 mM DTT; 0.5 mM PMSF and Complete protease inhibitors (Roche Applied Science, Penzberg, Germany) for 15 minutes. A sample was taken to provide total protein. Cytoplasmic proteins were released by vortexing in 4% NP-40 (Sigma-Aldrich, St. Louis, MO, USA) and collected in the supernatant after centrifugation for 30 seconds at 13500 g. The pellet was washed in cell swelling buffer three times and resuspended in three times the pellet volume of 20 mM HEPES pH7.9; 0.4 M NaCl; 1 mM EDTA; 1 mM EGTA; 1 mM DTT; 1 mM PMSF and protease inhibitors to release nuclear proteins. Nuclear proteins were collected from the supernatant after centrifugation for 5 minutes at 13500 g.

Western blotting was performed using the following primary antibodies; rabbit anti-Evc2 (R1656 described here); sheep anti-Evc (S43G,[7]), mouse anti-α tubulin (clone B-5-1-2, Sigma-Aldrich, St. Louis, MO, USA); mouse anti-β actin (clone AC-15, Sigma-Aldrich, St. Louis, MO, USA) and rabbit anti-c-Jun (60A8, Cell Signalling Technology, Beverly, MA, USA). Secondary antibodies were peroxidase-conjugated, donkey anti-sheep (Jackson ImmunoResearch Labs Inc; PA, USA); goat anti-mouse (Thermo Scientific, Walthem, MA, USA) and goat anti-rabbit (Jackson ImmunoResearch Labs Inc; PA, USA). Peroxidase was detected using the SuperSignal West Dura extended duration substrate (Thermo Scientific, Walthem, MA, USA).

## Authors' contributions

HJB drafted the manuscript and demonstrated that Evc2 is required for Hh signalling; that Evc2 localizes to the basal body; that Evc/Evc2 cilia localization is co-dependent and that Evc2 is found in the nucleus. ST performed the yeast-2-hybrid analysis and Co-IPs. Y-N L performed the TEM analysis. JC demonstrated that native Evc and Evc2 localize to MC3T3 cilia. KM performed luciferase assays. CP performed the bioinformatic analyses. VRP prepared the construct to create Evc2 null mice used to derive MEFs and assisted in the preparation of the manuscript. JG conceived the study, participated in the design of the experimental work and interpretation of findings and drafted the manuscript.

## Supplementary Material

Additional file 1 **A phylogenetic tree of *EVC *and *EVC2 *sequences**. The tree was constructed with the Fitch-Margoliash algorithm using a Poisson genetic distance and global optimization with bootstrapping. Bootstrap values at internal nodes not supported at 100% are provided. Species abbreviations: Bf, *Branchiostoma floridae*; Hs, *Homo sapiens*; Lg, *Lottia gigantea*; Mm, *Mus musculus*; Nv, *Nematostella vectensis*; T, *Trichoplax adhaerens*; Tr, *Tetraodon nigroviridis*; and, Xt, *Xenopus tropicalis*. Click here for file
