# Supplementary figures and images for: Evc2 is a positive modulator of Hedgehog signalling that interacts with Evc at the cilia membrane and is also found in the nucleus
Source: BMC Biol. 2011 Feb 28;9:14. doi: 10.1186/1741-7007-9-14 (PMC3052239; doi:10.1186/1741-7007-9-14)

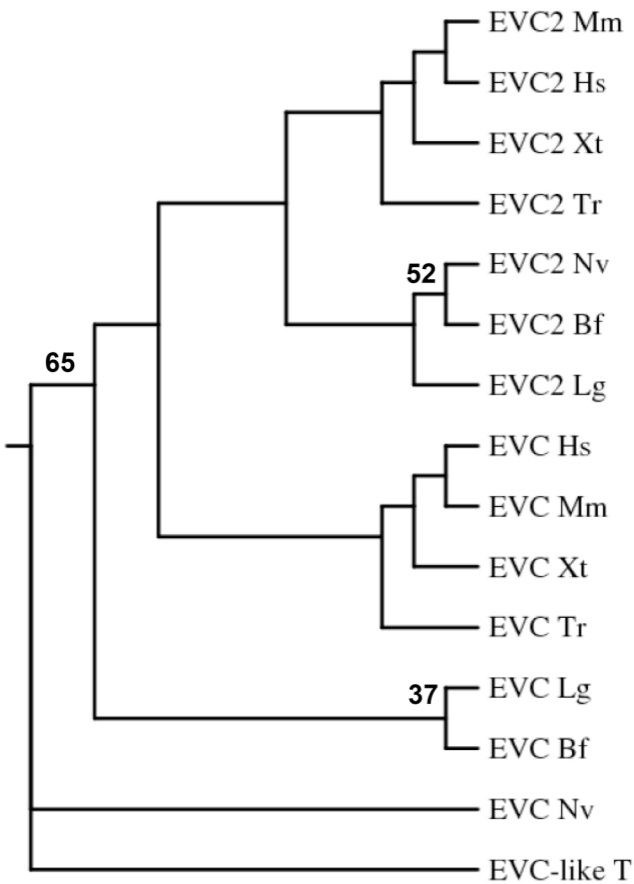

Supplement: Additional file 1 — A phylogenetic tree of EVC and EVC2 sequences. The tree was constructed with the Fitch-Margoliash algorithm using a Poisson genetic distance and global optimization with bootstrapping. Bootstrap values at internal nodes not supported at 100% are provided. Species abbreviations: Bf, Branchiostoma floridae; Hs, Homo sapiens; Lg, Lottia gigantea; Mm, Mus musculus; Nv, Nematostella vectensis; T, Trichoplax adhaerens; Tr, Tetraodon nigroviridis; and, Xt, Xenopus tropicalis. [file 1741-7007-9-14-S1.PDF]
